# Paternal and Maternal Genetic Analysis of a Desert Keriyan Population: Keriyans Are Not the Descendants of Guge Tibetans

**DOI:** 10.1371/journal.pone.0100479

**Published:** 2014-06-26

**Authors:** Kaixu Chen, Abdurahman Ablimit, Fengjun Ling, Weiwei Wu, Wenjuan Shan, Wenbei Qin, Tuerhong Keweier, Hongli Zuo, Fuchun Zhang, Zhenghai Ma, Xiufen Zheng

**Affiliations:** 1 Xinjiang Key Laboratory of Biological Resources and Genetic Engineering, College of Life Science and Technology, Xinjiang University, Urumqi, Xinjiang, China; 2 Beijing Entry-Exit Inspection and Quarantine Bureau, Beijing, China; 3 Institute of Forensic Science of Zhejiang, Hongzhou, China; 4 Blood Center of Urumqi, Xinjiang, China; 5 Department of Pathology, The University of Western Ontario, London, Canada; 6 Lawson Health Research Institute, London, Canada; University of Toronto, Canada

## Abstract

The Keriyan people live in an isolated village in the Taklimakan Desert in Xinjiang, Western China. The origin and migration of the Keriyans remains unclear. We studied paternal and maternal genetic variance through typing Y-STR loci and sequencing the complete control region of the mtDNA and compared them with other adjacent populations. Data show that the Keriyan have relatively low genetic diversity on both the paternal and maternal lineages and possess both European and Asian specific haplogroups, indicating Keriyan is an admixture population of West and East. There is a gender-bias in the extent of contribution from Europe vs. Asia to the Keriyan gene pool. Keriyans have more genetic affinity to Uyghurs than to Tibetans. The Keriyan are not the descendants of the Guge Tibetans.

## Introduction

The Taklimakan Desert located in the centre of the Tarim Basin in Western China is China’s second largest desert. There is an ancient isolated village along the Keriya River in the depth of the Desert. The village with more than 200 families and over 1000 individuals spans more than 200 kilometers from the south of the Keriyan River to the north. It is said they have lived there for more than 400 years. They call themselves as “Keriyan”. The village and Keriyans were first introduced to the world by a Swedish geographer and explorer Sven Anders Hedin when he explored Western China in1896. Due to its geographical location and traffic blockage, Keriyans still live isolated from the outside of the world, except for some essential commercial trade with adjacent areas. The village is surrounded by the desert and the living environment is very harsh. Life there is very simple and primitive. Keriyans speak a kind of language similar to the Uyghur language belonging to the Altai linguistic family. However, the origin and migration history of the Keriyan is unclear. There are three hypotheses about their ancestors: one is that Keriyan people are descendants of the ancient Guge, Tibetans who climbed through the Kunlun Mountains after the collapse of the ancient Guge Kingdom in 1630 and have lived in that area since then; The second argument is that they are originally Keriyan indigenous peoples, who resided there as early as the Neolithic Age; the third argument is that they are a branch of the ancient Loulan people who mysteriously disappeared 2000 years ago, the descendants of the Mi Dynasty in the Western Han. A finer patrilineal and matrilineal genetic investigation of Keriyans would help to dissect the issue.

In this study, we collected DNA samples from the Keriyan village and genotyped them with 17 Y-chromosomal short tandem repeats (STR) or microsatellite loci and the complete control region of the mitochondria DNA. Analyses of the genetic association with other reference populations were performed to dissect the origin of the Keriyan.

## Materials and Methods

### 1. DNA samples

**Figure 1:**
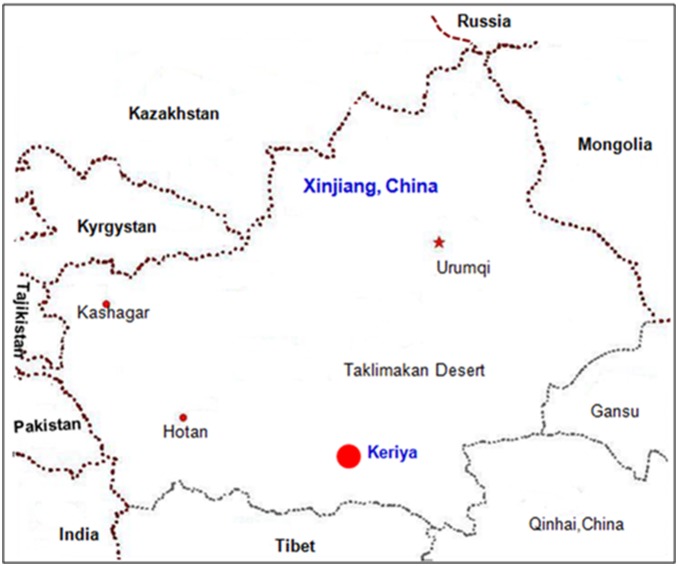
Geographic locations of the samples in this study.

A total of 70 blood samples were randomly collected from unrelated healthy individuals from the Keriyan village in Xinjiang (Figure 1) with written informed consents. The study was approved by the Ethnic Study Committee of Xinjiang University. DNA was extracted from the blood using the classic phenol/chloroform method. For comparative analysis, we examined information on haplotypes of Y-STR loci and mtDNA sequence. The reference population data for Y-STR genotyping included: Archangelsk, Russian Federation [Russian], population sample with 42 haplotypes(YA003171); Amdo, China [Tibetan], population sample with 88 haplotypes(YA003694); Kham, China [Tibetan], population sample with 109 haplotypes(YA003695); Liaoning, China [Mongolian], population sample with 206 haplotypes(YA003758); Liaoning, China [Northern Han], population sample with 376 haplotypes (YA003756); Central Mongolia [Khalkh], population sample with 87 haplotypes (YA003737); Northern Mongolia [Khalkh, Darkhad, Uriankhai] population sample with 29 haplotypes (YA003734); Rjasan, Russian Federation [Russian], population sample with 36 haplotypes (YA003178); Wallachia, Romania [Romanian], population sample with 96 haplotypes (YA003457); Western Mongolia [Khalkh, Durvud, Bayad, Uriankhai, Khazak, Torguud, Uuld, Zakhchin], population sample with 33 haplotypes (YA003732); Xinjiang, China [Uyghur], population sample with 218 haplotypes (YA003847); Kazakhstan, population set of 3 population samples with 347 haplotypes (YHRD database); Mongolia, population set of 9 population samples with 445 haplotypes (YHRD database); Romania, population set of 14 population samples with 817 haplotypes (YA003457); and Russian Federation, population set of 50 population samples with 3364 haplotypes (YHRD database).

For the mtDNA data, reference populations included Kazakhs, Kirghiz, Uzbeks and Uyghurs [1], [2]; Mongolians [2]–[5]; Daurs, Ewenkis and Oroqens [4] [6]; Huis [2]; Armenians, Azeris and Georgians [7]; Tuvans and Buryats [8]; Northern and Southern Hans [4], [9], [10], and Southern East China ethic groups [11], [12], Tibetan [12], [13], Korean [4], [14], Japanese [15], Russian and Siberian [16], [17]. We also included our own collected data from Uyghurs from Xinjiang China (unpublished).

### 2. Y-STR genotyping

Genotyping of 17 Y-STR loci (DYS 19, 389I, 389II, 390, 391,392,393, 385I, 385II, 437, 438, 439, 448, 456, 458, 635 and GATAH4) in Keriyans was conducted with an Applied Biosystems (ABI) AmpFlSTR Yfiler PCR amplification kit (ABI, Carlsbad, CA) and analysed by an Applied Biosystems 3100 DNA sequencer (ABI), according to the manufacturer’s instruction. Genotypes were identified based on the number of tandem repeats.

The Keriyan samples were classified into haplotypes based on their genotypes summarized from the 17 Y-STR loci and further assigned into haplogroups using an online haplogroup predictor tool (http://www.hprg.com/hapest5).

### 3. mtDNA sequencing and sequence comparison

The control regions of the mtDNA were amplified using the primer set L15996/H601 as described previously [18], [19]. PCR product was purified with a fragment DNA/RNA purification kit (Biomiga, Beijing, China) and used as a DNA template for DNA sequencing. Cycling sequencing for HVS I and HVS II was conducted using the ABI BigDye terminator v3.1 cycle sequencing kit in an ABI 3100 DNA sequencer.

Nucleotide sequences were edited and aligned using the MEGA 5.0 (DNAStar, Inc.) [20], and compared with the Revised Cambridge Reference Sequence (rCRS) [21]. mtDNA haplogroups were assigned using online tool HaploGrep (http://www.haplogrep.uibk.ac.at) with the strategy described by Yao et al [22].

### 4. Data analyses

The frequencies of Y-STR haplotypes and of mtDNA haplogroups of the Keriyan samples were estimated. The basic parameters of molecular diversity (nucleotide diversity, haplotype diversity, and mean number of pairwise differences) and population genetic structure (including analyses of molecular variance, AMOVA), neutrality tests (Tajima’s *D* and Fu’s *F*_s_), and genetic distance *Fs*t were estimated for mtDNA using the computer program Arlequin 3.11 (http://www.cmpg.unibe.ch/software/arlequin3. Computational and Molecular Population Genetics Lab, Institute of Zoology, University of Berne, Baltzerstrasse 6, 3012 Bern, Switzerland). The statistical significance of *Fst*-values was estimated by permutation analysis using 10000 permutations. Neighbor joining tree was constructed using Fst values by Phylip v3.6.9 (http://evolution.genetics.washington.edu/phylip.html).

Using an AMOVA tool provided by the YHRD website (http://www.yhrd.org/Analyse), genetic distances (*Rst*) between the Keriyan population and other reference populations for the Y-STR data set were analyzed and *p* values were calculated with 10000 permutations using *R_st_* between populations. A multiple dimension scaling plot (MDS) was obtained using *Rst* generated by AMOVA. Median-joining (MJ) networks were constructed with the Network 4. 6.12 program (http://www.fluxus-engineering.com) using the HVRI sequences or Y-STR genotypes [23], [24]. All polymorphic nucleotide positions were assigned weights following the recommendations provided by Roostalu et al [25]. The Y-STR loci were weighted based on the inverse of their variances.

To compare mtDNA variation among Keriyan and the surrounding other populations, a principal component analysis based on haplogroup frequencies among populations was performed using SSPS version 17 software.

In addition, the coalescence times of haplogroups M7c, M8a, and M9ab, and M11 were computed using rho statistics [26] calibrated with two different mutation rates, including: i) the most widely used mutation rate of one transitional step between nps 16090–16365 every 20,180 years [26] and ii) the revised rate of one mutation every 18,845 years for the same sequence region [27]. The standard deviation for the rho estimates was obtained according to Saillard et al [28], and the length variation in the polycytosine tract between nps 16180–16193 was excluded from the analysis.

## Results

### 1. Genetic diversity of Y-chromosomal markers

**Figure 2:**
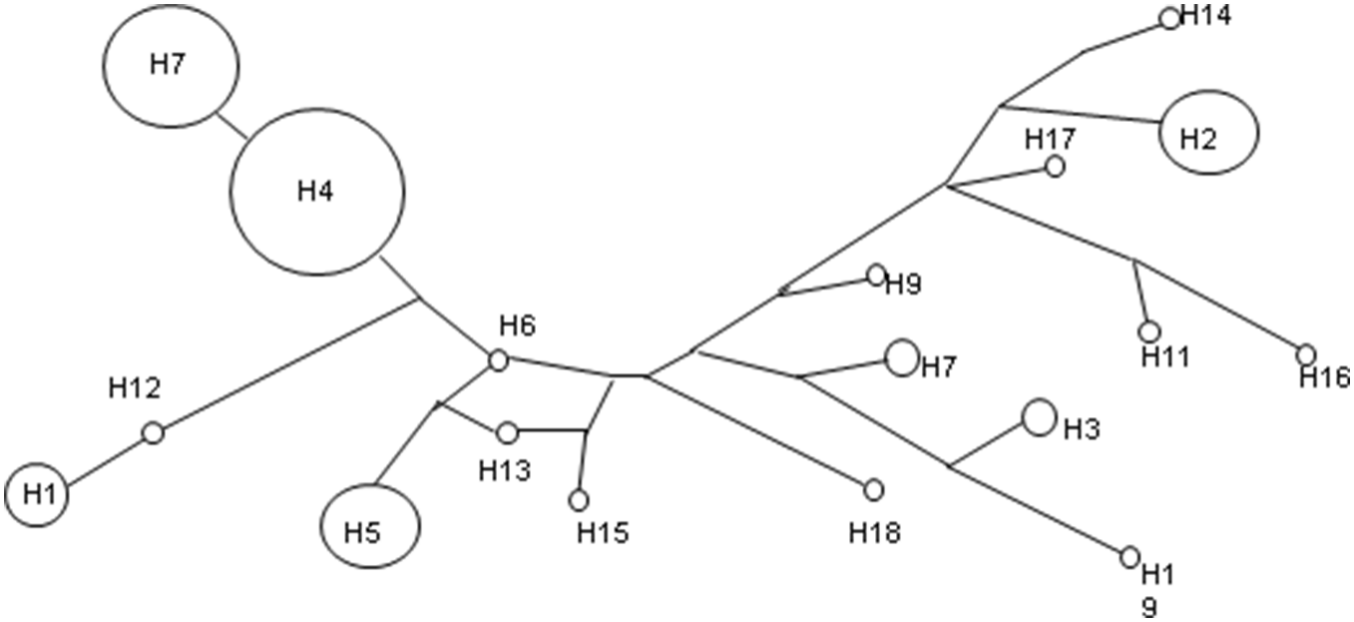
Medium Network of Keriyans using haplotypes from 13 Y-STR loci. A Median Joining network was constructed using 13 Y-STR loci (DYS389I, 390, 391, 392, 393, 437, 438, 439, 448, 456, 458, 635 and GATAH4) as described in the Materials & Methods. Node size is proportional to frequencies of haplotype.

We detected 17 Y-STR loci using Applied Biosystems AmpFlSTR Yfiler. A total of 20 haplotypes were observed in the 50 Keriyan male samples. The gene diversity was 0.896. We also designed these haplotypes into 9 haplogroups according to their genotypes using online haplogroup predictor tool (http://www.hprg.com/hapest5). The frequencies and genotypes of haplotypes are summarized in Table 1. Five haplotypes were observed more than twice in the Keriyans while 13 haplotypes were observed once and two haplotypes were observed twice. The most common haplotype was H4 belonging to haplogroup R1a, shared by 13 people at the frequency of 26%. More than 60% of samples belong to Haplogroup R1a. The European- specific haplogroups R1a, J and I account for 82% of the total haplogroups, whereas Asian-specific haplogroups H, L and T for 10%. Central Asian or Eurasian specific haplogroup Q and N account for 8%. A network of Y-STR haplotype was constructed for the Keriyan samples (Figure 2). The stretched, not star-like network showed that the Keriyan samples had at least two ancestry haplotypes.

**Table 1:** Haplotypes of 17 Y-STR loci and their frequencies in the Keriyans.

Haplotype	Haplogroup	DYS19	DYS385	DYS389I	DYS389II	DYS390	DYS391	DYS392	DYS393	DYS437	DYS438	DYS439	DYS448	DYS456	DYS458	DYS635	YGATAH4	Frequency
H1	Q	14	13,21	15	30	23	10	10	15	14	11	12	19	16	17	27	11	0.06
H2	J2a1b	14	13,16	13	30	22	11	11	12	15	9	11	21	16	15	22	11	0.1
H3	J1	14	11,17	13	29	23	10	13	12	14	10	11	20	14	18.2	23	10	0.04
H4	R1a	16	11,14	13	29	25	10	12	13	14	11	10	20	16	16	24	11	0.26
H5	R1a	16	11,14	13	31	25	10	11	13	14	11	10	20	16	15	25	13	0.1
H6	I2a	16	12,12	13	29	24	9	11	13	14	10	11	20	15	18	23	10	0.04
H7	R1a	16	11,14	13	29	26	10	12	13	14	11	10	20	16	16	24	11	0.14
H8	J2a1b	14	12,16	13	30	22	11	11	12	15	9	11	21	16	15	22	11	0.02
H9	H	16	15,16	13	29	21	10	11	12	14	8	11	18	16	16	21	11	0.02
H10	R1a	17	11,14	14	32	25	11	11	13	14	11	10	20	15	18	23	13	0.02
H11	N	14	11,13	13	30	23	10	14	14	14	10	10	19	14	19	22	11	0.02
H12	L	14	12,21	13	29	24	10	14	13	16	11	10	20	15	18	20	12	0.02
H13	L	14	13,18	14	30	26	11	13	12	15	10	12	20	16	17	21	11	0.02
H14	L	14	13,21	15	30	23	10	10	15	14	11	12	19	16	17	26	11	0.02
H15	R1a	15	11,14	13	31	25	10	11	13	14	11	10	20	16	15	25	13	0.02
H16	R1a	15	11,14	14	33	25	10	11	13	14	11	10	20	15	15	23	13	0.02
H17	R1a	15	12,13	13	30	24	10	11	13	14	9	10	19	14	18	20	12	0.02
H18	T	15	14,25	13	30	24	11	13	13	15	10	12	20	15	16	20	11	0.02
H19	R1a	16	11,14	13	29	25	10	11	13	14	11	10	20	16	16	23	12	0.02
H20	J2a1b	14	11,16	13	28	24	10	11	12	16	9	11	19	15	16	21	11	0.02

We searched all 20 haplotypes observed in the Keriyan in the YHRD database comprising of 71234 haplotypes from 477 populations. Three haplotypes (H2, H6 and H19) matched certain samples in the database. Haplotype 2 (J2a1b) representing at the frequency of 10% in the Keriyan was found to match one haplotype in the searching database. The hit haplotype belonging to Eurasian Met population database (32785 matching haplotypes in 221 populations) is from Xinjiang Uyghur population reported by our group (YA003847) [29]. Haplotype 6 occurring twice in the Keriyan population was found to have 4 matching haplotypes in the searched database. Three of them were found in the Eurasian metapopulations, among which two matched the Central Mongolia Khalkh (YA003737) and one matched the Mongolian population from Liaoning, China (YA003758), respectively. The other hit was in the Northern Han population from Liaoning, China (YA003756). Moreover, Haplotype 19 occurring once in the Keriyan population was found in the Croatian of Zagreb, Croatia dataset in Eurasian-European-South Eastern European database (YA003130).

### 2. Patrilineage affinity of the keriyan population

**Figure 3:**
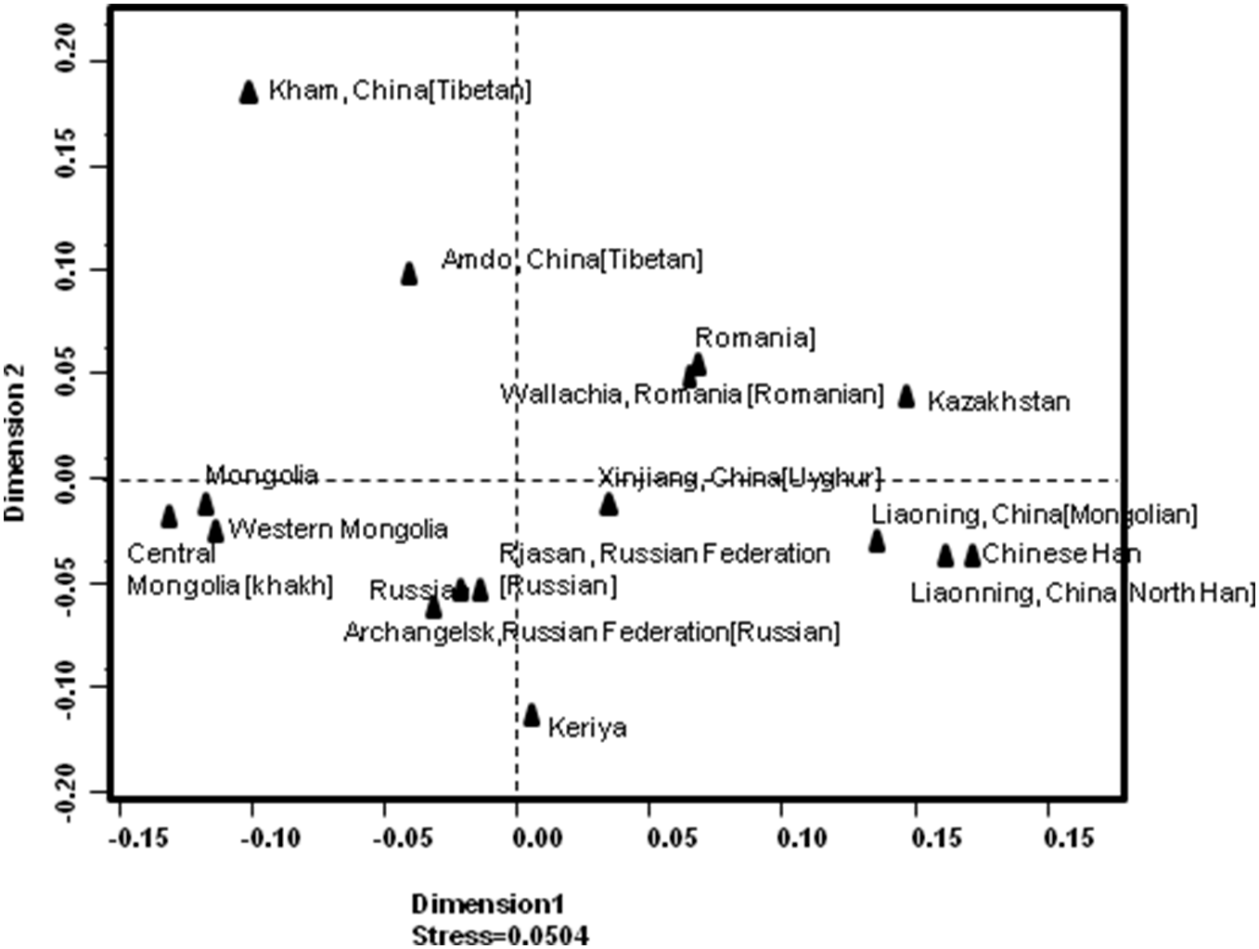
Multiple dimension Scaling plots of *Rst* value estimated from haplotypes comprised 17 Y-STR loci. *Rst* values were calculated between each populations and MDS plot based on *Rst* using an online tool AMOVA provided by YHRD.

In order to know the genetic relationship of the Keriyan samples with other populations, we performed an AMOVA analysis and calculated *Rst* with the genotype of 17 Y-STR loci from the Keriyan and other 11 populations and 4 population sets consisting of a total of 6314 haplotypes published in the YHRD database. The obtained *Rst* is shown in Table S1. To visualize the extent of genetic diversity among these populations, a MDS plot based on *Rst* was made as shown in Figure 3. Not surprisingly, the MDS plot clearly showed a geographic affinity pattern. For instance, Chinese mainland Hans including north Han and Beijing Han, China populations and north-China Mongolian clustered together; east European populations from Russia and Siberia gathered together and formed a cluster; Mongolia populations were close to each other, and Romania populations gather together and formed their own cluster. Two Tibetan populations did not form a close cluster like the above populations, but distinctively separated from the Keriyan and the other populations. The Keriyan population was not attached to any other populations. However, the Keriyan population was relatively closer to the Russia populations and Xinjiang Uyghur than to Mongolia, Romania, China north Mongolian and Han, and Tibetan populations. The greatest distances between the Keriyan samples were populations from the Southern Chinese Han and Tibetan, followed by Mongolians. Even so, the distance between the Keriyans and the Russian, or Siberians, or Uyghur was significant.

**Figure 4:**
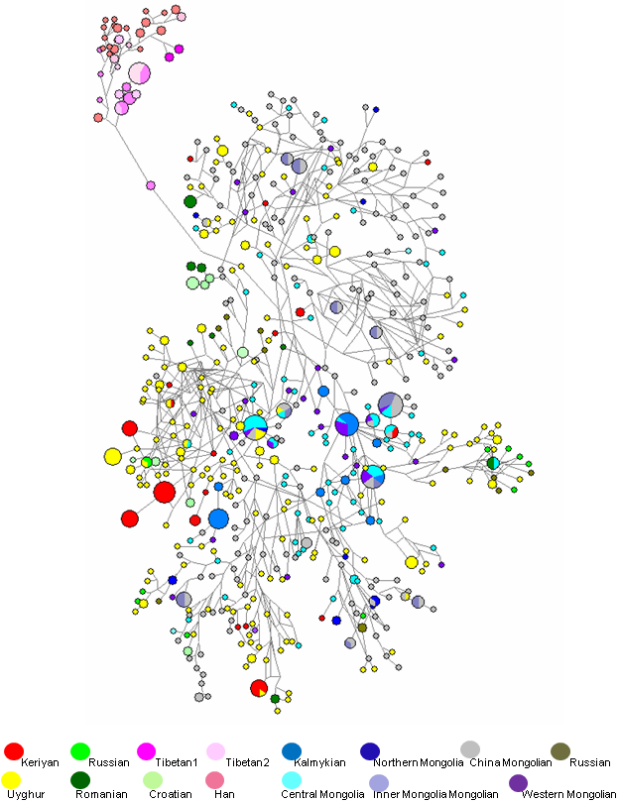
Median Joining network of Keriyans and other 14 referenced populations using 13 Y-STR haplotypes. A Median Joining network was constructed using the same 13 Y-STR loci as Figure 2. Node size is proportional to frequencies of haplotype. Populations were labelled by different colors.

In order to visualize the phylogenetic relationship between Keriyan and other populations, we constructed a MJ network. We used the haplotypes from13 Y-STR loci (DYS 389I, 390, 391, 392, 393, 437, 438, 439, 448, 456, 458, 635 and GATAH4) in making a network due to mutations occurring at DYS19, 389II and 385. The reference populations were selected based on the genetic affinity between Keriyan and the other populations based on the MDS analysis (Figure 3). Only the part of the common haplotypes from selected populations listed on YHRD was included to construct MJ network of Y-STR due to limitations in the software of the Network 4.6.12 program. As shown in Figure 4, the haplotypes from the Keriyan were generally close to European and Mongolian populations, but far away from Sino-Tibetan populations (Tibetan and Han populations). Two thirds of Keriyan haplotypes gathered together with the European populations Russian and Siberia and Eurasian Uyghur population, while the remainder gathered with Mongolian populations.

### 3. Maternal analysis

We sequenced the complete control region of the mtDNA for 42 samples from the Keriyan. Considering both hyper variable segments (1040 bp), a total of 59 positions were mutant. The average number of nucleotide differences (k) was 9.29568. Nucleotide diversity (Pi) and haplotype (gene) diversity Hd were 0.00832 and 0.947, respectively. Due to lack of HVS II sequence information for some populations, we just selected HVS I sequences to compare molecular diversity among populations. Shown in Table 2, the Keriyan have the lowest gene and nucleotide diversity. We also calculated the neutrality indexes, Tajima’s *D* and Fu’s *Fs* to examine whether there is a population expansion. Tajima *D* and Fu’s *F* values were −1.13 and −1.157, respectively, but both were not significant (*P*>0.05), indicating more recent but not historical population expansion.

**Table 2:** Gene diversity of Keriyan and its surrounding populations.

Population	No. ofsamples	No. of differentsequences	Genediversity	Nucleotidediversity
Keriyan	42	13	0.896±0.022	0.010±0.0056
Daheyan^a^	58	27	0.949±0.015	0.0188±0.010
Uzbek^b^	58	51	0.995±0.005	0.0189±0.010
Uyghur^b^	47	42	0.995±0.006	0.019±0.011
Kazak^b^	53	50	0.998±0.004	0.021±0.011
Mongolian^b^	48	38	0.989±0.007	0.017±0.001
Hui^b^	44	41	0.997±0.006	0.0197±0.010
Tivan^c^	96	50	0.971±0.008	0.0163±0.009
R-Tibetan^d^	57	39	0.969±0.014	0.0167±0.009
G-Tibetan^d^	43	41	0.998±0.006	0.0207±0.011
S-Tibetan^d^	44	40	0.996±0.006	0.0228±0.012
Q-Tibetan^d^	67	56	0.992±0.005	0.0219±0.011
Daur^e^	45	30	0.979±0.009	0.016±0.001
Ewenki^e^	47	21	0.956±0.011	0.017±0.001
Oroqen^e^	44	21	0.948±0.015	0.016±0.001
Han^f^	47	42	0.993±0.007	0.0194±0.010
Russian^g^	267	153	0.965±0.008	0.0152±0.008
Tatar^h^	73	56	0.991±0.004	0.0153±0.008

a : Zhou H et al 2010;

b : Yao Y et al 2004;

c : Starikovskaya E et al 2003;

d : Zhao M et al 2009;

e : Kong Q et al 2003;

f : Yao Y et al 2002;

g : Gokcumen O et al 2008;

h : Malyarchuk B et al 2010.

**Figure 5:**
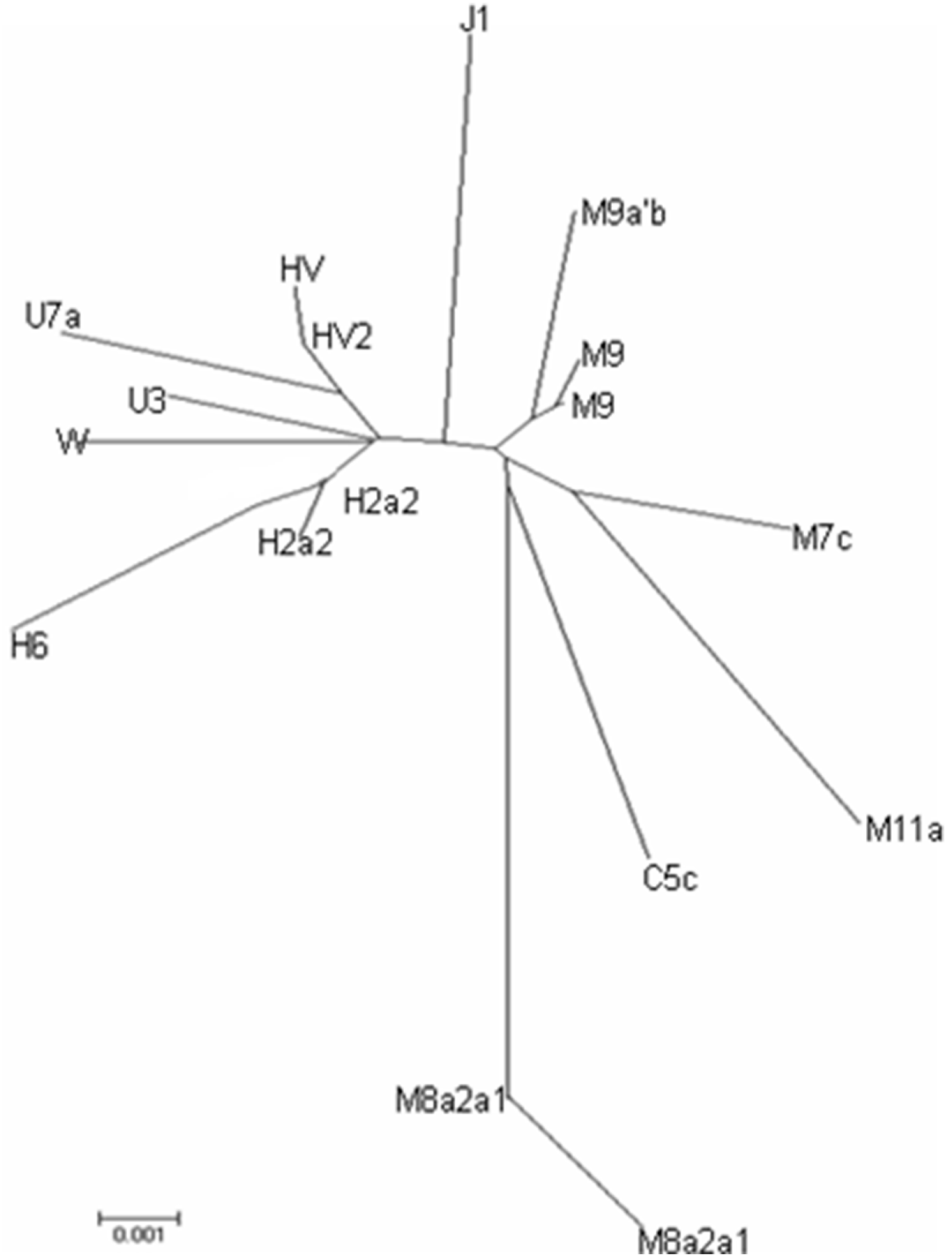
A Neighbour-Joining tree for the Keriyans based on the control region sequences of mtDNA.

We observed a total of 20 different sequences (haplotypes) and assigned them into 13 different sub-haplogroups (Table S2). The nucleotide mutations of each haplotype and the frequencies of each haplogroup are listed in Table S2. A Neighbour Joining Tree (Figure 5) was constructed using haplotypes from the Keriyan samples. The Keriyan population consists both of Europe-specific lineages (HV, H2a2, H6, J1, U3, U7a, and W) and Asian-specific-lineages (C5c, M7c, M8a2a1, M9 and M11a) as shown in Figure 5 and Table S2. The percentages of West Eurasian and East Eurasian specific lineages were equal, 50% and 50%, respectively. The most dominant haplogroup was HV with a frequency of 19.05%, followed by sub-Hg H2a2 and M11a both with a frequency of 14.29%. Except for C5c, all other East Asian specific haplotypes were represented more than twice in the Keriyan population, while the majority of European specific haplogroups (H6, J1, U3 and U7a) except H2a2, HV2 and W appeared only once in the population. We merged sub-haplogroups into a major haplogroup in order to compare with other reference populations. The frequencies of the final merged haplogroups were shown in Table 3.

**Table 3:** Frequencies of haplogroup in the Keriyan and referenced populations.

Haplogroup	A	B	C	D	F	G	H	HV	I	J	K	M	N	S	R	T	U	V	W	X	Y	Z	Other
Russian	0	0.625	12.5	20	3.75	8.75	0	0	0	0	0	7.500	4.375	0	0	0	0	0	0	0	43.125	0.625	0
daheyan	5.20	3.50	1.70	3.50	3.50	3.50	24.10	1.70	0.00	0.00	0.00	24.10	3.50	0.00	6.90	3.50	5.20	0.00	0.00	8.60	0.00	1.70	0.00
keriyan	0.00	0.00	2.38	0.00	0.00	0.00	16.67	19.05	0.00	2.38	0.00	47.62	0.00	0.00	0.00	0.00	4.76	0.00	7.14	0.00	0.00	0.00	0.00
MIMg	11.61	7.75	9.04	25.17	13.55	5.16	0.00	3.23	0.65	1.94	1.94	14.22	1.94	0.00	2.59	0.00	0.65	0.00	0.00	0.00	0.00	0.65	0.00
MYMg	6.52	13.05	8.69	17.38	8.70	8.69	0.00	0.00	0.00	0.00	0.00	13.04	2.17	0.00	13.04	0.00	0.00	0.00	0.00	0.00	0.00	8.70	0.00
MMg	4.85	10.68	13.59	24.28	8.74	5.83	0.00	4.85	0.00	0.00	0.97	10.67	1.94	0.00	3.88	1.94	2.91	0.00	0.00	0.00	0.97	3.88	0.00
MEMg	8.33	10.42	6.25	39.58	8.33	4.17	0.00	0.00	0.00	0.00	0.00	8.33	6.25	0.00	2.08	2.08	0.00	0.00	0.00	0.00	0.00	4.17	0.00
MWMg	3.37	2.24	16.85	31.44	6.74	6.74	5.62	0.00	0.00	6.74	3.37	7.86	1.12	0.00	0.00	2.25	1.12	0.00	1.12	0.00	0.00	3.37	0.00
YLMg	8.20	2.04	10.20	20.44	18.37	16.33	4.12	2.00	0.00	0.00	0.00	8.16	2.04	0.00	0.00	0.00	8.16	0.00	0.00	0.00	0.00	0.00	0.00
Kaz	3.77	3.77	13.21	13.21	7.55	5.66	7.55	5.66	1.89	1.89	0.00	9.43	1.89	0.00	0.00	7.55	3.77	1.89	0.00	0.00	0.00	11.32	0.00
Uzb	3.45	5.17	1.72	22.41	8.62	6.90	10.35	1.72	0.00	1.72	1.72	3.45	6.90	0.00	1.72	3.45	13.79	0.00	0.00	3.45	1.72	1.72	0.00
Uyg	4.26	2.13	6.38	10.64	6.38	12.77	10.64	4.26	0.00	4.26	2.13	14.89	0.00	0.00	0.00	2.13	12.77	0.00	6.38	0.00	0.00	0.00	0.00
Hui	6.67	17.78	2.22	15.56	13.33	8.89	0.00	0.00	0.00	0.00	0.00	24.44	0.00	0.00	2.22	4.44	0.00	0.00	0.00	0.00	0.00	4.44	0.00
NH	8.01	11.04	3.25	25.10	11.48	4.55	0.00	0.00	0.00	0.00	0.00	23.16	5.62	0.00	1.95	0.00	0.00	0.00	0.00	0.00	1.95	2.38	1.51
SH	6.28	19.67	3.74	15.79	15.12	3.40	0.00	0.00	0.00	0.00	0.00	22.76	6.45	0.00	1.87	0.00	0.00	0.00	0.00	0.00	0.51	4.24	0.17
Dau	2.22	15.56	6.67	24.44	0.00	8.89	0.00	0.00	0.00	2.22	0.00	20.00	2.22	0.00	4.44	2.22	0.00	0.00	0.00	0.00	2.22	8.89	0.00
Ewe	4.26	10.64	19.15	31.91	2.13	8.51	8.51	0.00	0.00	10.64	0.00	0.00	0.00	0.00	0.00	0.00	0.00	0.00	0.00	0.00	0.00	4.26	0.00
Oro	4.55	2.27	29.55	43.18	4.55	11.36	0.00	0.00	0.00	0.00	0.00	0.00	2.27	0.00	0.00	0.00	0.00	0.00	0.00	0.00	0.00	2.27	0.00
Arm	0.00	0.00	0.00	0.00	0.00	0.00	20.00	6.70	0.00	16.70	6.70	0.00	3.30	0.00	0.00	10.00	26.70	0.00	0.00	10.00	0.00	0.00	0.00
Geo	0.00	0.00	0.00	0.00	0.00	0.00	17.90	7.10	0.00	3.60	7.10	0.00	7.10	0.00	10.70	10.70	28.60	0.00	0.00	7.10	0.00	0.00	0.00
TV	2.10	8.50	43.20	13.80	4.20	10.60	4.20	0.00	0.00	2.10	0.00	1.60	3.20	0.00	0.00	0.00	3.20	0.00	2.10	0.00	0.00	1.60	0.00
BR	0.00	4.00	40.00	16.00	0.00	8.00	4.00	0.00	0.00	4.00	0.00	0.00	0.00	4.00	0.00	0.00	12.00	4.00	0.00	0.00	0.00	4.00	0.00
UYG2	4.41	0.00	7.35	12.75	3.43	7.35	16.67	2.94	0.98	3.92	0.49	11.76	2.45		1.96	4.90	9.31	0.00	3.92	0.00	2.45	1.96	0.00
Nakchu_ Tibetan	12.50	4.76	1.79	27.38	11.90	8.93	0.00	0.00	0.00	0.00	0.00	31.55	1.79	0.00	0.60	0.00	0.60	0.00	0.00	0.00	0.00	2.98	0.00
Shigatse_ Tibetan	14.55	1.36	7.27	22.27	12.73	6.36	0.00	0.45	1.36	0.00	0.00	38.18	0.45	0.00	1.82	0.00	0.45	0.00	0.00	0.00	0.00	0.91	0.00
Tibet_ Tibetan	11.11	5.56	3.70	20.37	16.67	12.96	0.00	0.00	0.00	0.00	0.00	29.63	3.70	0.00	0.00	0.00	0.00	0.00	0.00	0.00	0.00	0.00	0.00
Qinghai_ Tibetan_1	19.64	1.79	7.14	21.43	3.57	10.71	0.00	0.00	0.00	0.00	0.00	37.50	1.79	0.00	1.79	0.00	1.79	0.00	0.00	0.00	0.00	0.00	0.00
Yunnan_ Tibetan_1	11.43	17.14	5.71	14.29	2.86	11.43	0.00	0.00	0.00	0.00	0.00	42.86	0.00	0.00	0.00	0.00	0.00	0.00	0.00	0.00	0.00	2.86	0.00
Yunnan_ Tibetan_2	25.00	0.00	0.00	25.00	8.33	4.17	0.00	0.00	0.00	0.00	0.00	37.50	0.00	0.00	0.00	0.00	0.00	0.00	0.00	0.00	0.00	25.00	0.00
Gansu-Tibetan	8.43	7.23	8.43	13.25	12.05	9.64	0.00	0.00	1.20	0.00	0.00	28.91	2.41	0.00	1.20	0.00	1.20	0.00	0.00	0.00	0.00	3.61	0.00

**Figure 6:**
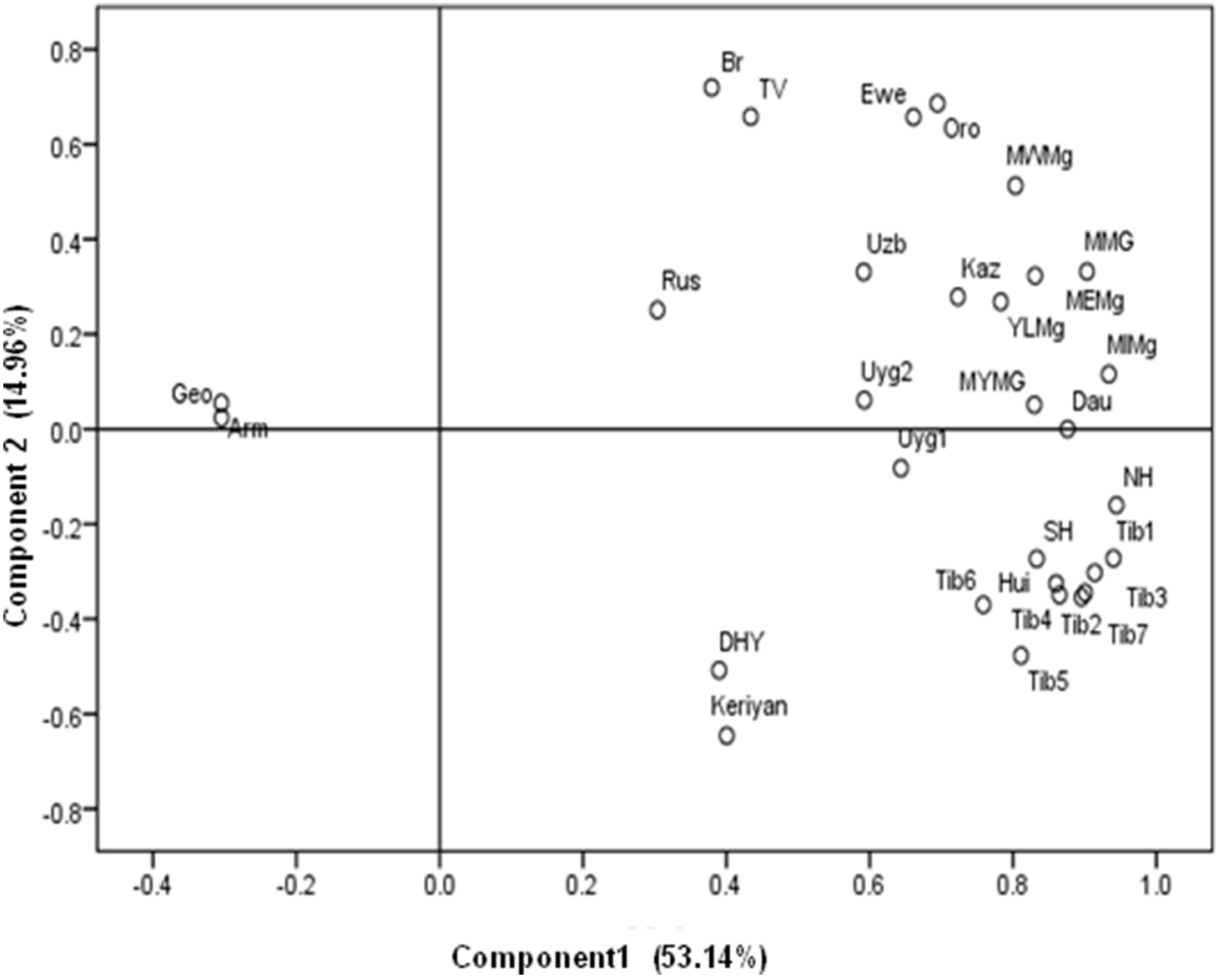
Phylogenetic relationship between Keriyan and reference populations analyzed by principal component analysis with the frequencies of haplogroups. Populations were coded as: MMg- Mongolians from Mongolia [3]; MWMg- Mongolians from Western Mongolia [5]; MEMg - Mongolians from east Inner Mongolia of China [4]; MIMg- Mongolians from central Inner Mongolia; MYMg - Mongolians from Yunnan Province of China [6]; YLMg- Mongolians from Yili; Uyg1 - Xinjiang Uyghurs from the Yao’s Study; Uyg2- Xinjiang Uyghurs from our unpublished data; Kaz - Kazaks; Uzb – Uzbeks [2]; Dau - Daurs; Ewe-Ewenkis; Oro- Oroqens; Arm- Armenians; Geo- Georgians from Cauasus [7]; TV- Tuvans; BR- Buryats from the south extreme of Siberia [8]; SH - South Han; NH- North Han [10]; DHY- Daheyan; Rus - Russian; Tib1- Nakchu; Tib 2- Tibetans from Shigats; Tib 3- Tibetans from Tibet; Tib 4- Tibetans from Qinghai; Tib5- Tibetans from Yunnan; Tib 6- Tibetans from Yunnan; Tib 7- Tibetans from Gansu.

To understand the genetic relationship of Keriyan population with other surrounding populations and to visualize the difference in the haplogroups among the populations, we performed a principal component analysis (PCA) using the basal frequencies of haplogroups of the Keriyan population in the study and the other 29 previously published populations as input vectors (Table 3). Figure 6 shows a PCA plot for the first two PCs, which account for 53.14%, and 14.96% of the total variance, respectively. In the PCA plot, the first two PCs revealed the following five distinguishing clusters: 1) The first one is comprised of the seven Tibetan groups and two Chinese Han groups at the right bottom corner of the plot; 2) a white population cluster including the Armenian and Georgia groups in the left of the plot; 3) a cluster comprising Xinjiang Eurasians, and the north China minority ethnic groups occupying the right upper corner; 4) a Russian cluster close to Siberia populations; and 5) the Keriyan and Daheyan populations. The Keriyan is placed very close to the Daheyan without any distance in the first component, but clearly separated from the other groups. The next closest to the Keriyan populations are the Uyghur, then Russian and Siberian populations, whereas the Keriyan is far away from the Tibetans.

To understand the genetic structure of Keriyan, we performed an AMOVA analysis and calculated *Fst*. To visualize the extent of genetic diversity among these populations, we plotted pairwise *Fst* values estimated from HVS I sequence data through MDS analysis (data not shown). Not surprisingly, Keriyans clustered together with the Daheyan group, with which they showed a similar haplogroup composition. The next closest to the Keriyan is the Uyghurs. By contrast, the Keriyans were quite genetically distinct from Tibetan, Han populations.

**Figure 7:**
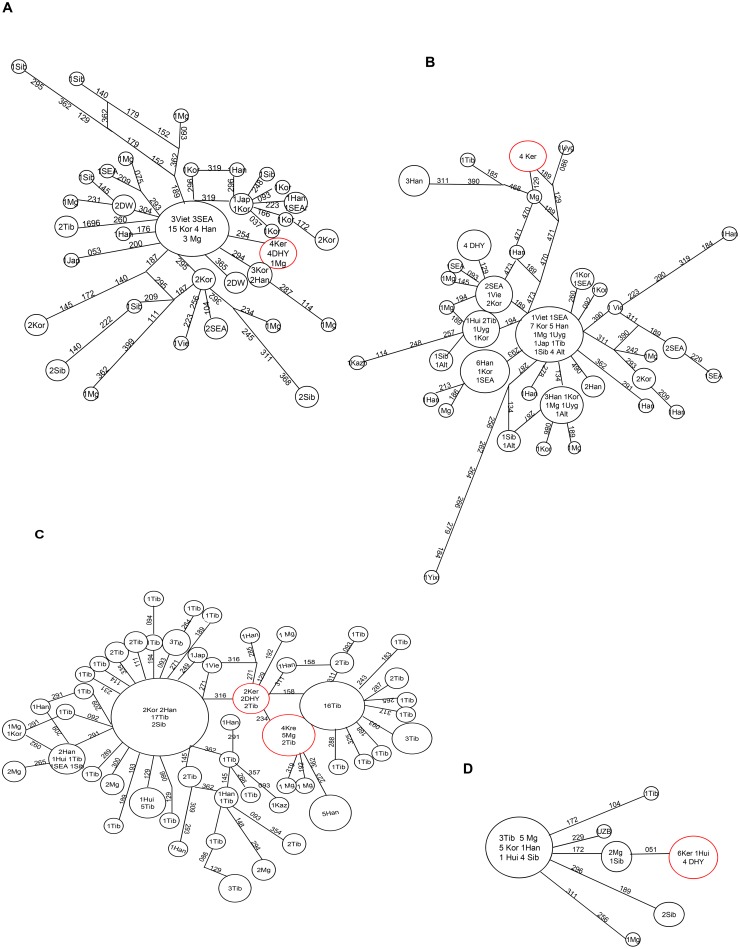
Reduced Median joining network of haplogroups M7c, M8a, M9 and M11 based on the mutation site of HVS I. The size of the circles is proportional to the number of sequences. Populations are coded as Mg- Mongolians; Sib- Siberia; SEA- South East Asian populations; Uyg1 - Xinjiang Uygurs; Kaz- Kazaks; Kor- Korean; Viet- Vietenese; Uzb – Uzbeks [2]; Dau - Daurs; Ewe- Ewenkis; Oro- Oroqens; Han- Chinese Han; DHY- Daheyan; Rus - Russian; Tib- Tibetans. Mutated sites (minus16, 000) were indicated along the lines. (A). Haplogroup M7c; (B). Haplogroup M8a; (C). Haplogroup M9; (D). Haplogroup M11.

In order to trace the origin of common Asian haplogroups of Keriyan, we constructed common haplogroups M7c, M8a, M9, and M11 networks using the Network 4.6.12 program as shown in Figures 7A–D, respectively. Overall, we observed that: 1) Each haplogroup displays as a star-like expansion cluster, indicating the ancestral haplotype expansion, 2) The Keriyan samples are not at the their respective central nodes of M7c, M8a, M9 and M11, implying these haplogroups do not exist in the Keriyan population for long time; and 3) Most of the Keriyan haplotypes are shared with Daheyan, except haplogroup M8a. Afterwards, we looked closer at haplotype differentiation within each haplogroup. M7c specifically found in East Asia [16], [30]–[32] has not been found in north eastern Asia [16], [33], [34] and is very rare in central Asians [35], [36], but was observed both in the Keriyan in this study and the Daheyan group reported by Zhou group [37] at the frequencies of 9.5% and 6.9, respectively. The Keriyans along with the Daheyans and one Mongolian are at the branch node with a step mutation at nucleotide position16254 away from the ancestral M7c characterized by 16223-6254-16295. The coalescence time of M7c in the Keriyan is ∼4037 years. In contrast to M7c, the Keriyan is at the very far end of the branch of the M8a network with multiple mutations away from the ancestral haplotype, but only one step away from a Uyghur with a mutation at position16189 or a Mongolian with a mutation at position 16129. M8a arose in the Keriyan an estimated 4036 years ago. It seems to have a very short history compared with the ancestral M8a which occurred in southern Asia 34437 years ago. M9 lineage which is prevalent with the greatest diversity in Tibetan, also exists in the Keriyan. The frequency of the M9 peaks in the Keriyans as compared with all other observed Asian specific haplogroups. In the Keriyans, there were two sub-haplogroups of M9 and M9a’b. The two Keriyans of sub-haplogroup M9 are at the same node with the two Daheyan samples and two Tibetans, while four Keriyans of M9a’b are shared by five Mongolian and two Tibetans with a back mutation. With regard to M11, all the six Keriyan of M11 and the four of Daheyan individuals are at the end of the same branch of M11. There are two step mutations at positions 16172 and 16051 differing to the ancestral root. The sub-haplogroup M11 seems to occur in the Keriyan and Daheyan groups very lately with an estimated coalescence time of 4324 years.

## Discussion

The Taklimakan Desert is located in the Tarim Basin which lies between the European and Asian Continents. Populations in this area have undergone substantial migrations and assimilations by other tribes during prehistoric and historic periods. The origin and migration history of populations in this area have drawn great attention. The archaeological and anthropological reports on the remains from the excavated tombs show that human beings inhabited the desert area in the Bronze age, approximately 4000 years BP and those found mummies display Eurasian features and belong to a West and East admixture population in genetics [38]. Data from extant people in the Daheyan village in the Taklimakan Desert has also verified this according to mtDNA sequence [37]. In this study, we for the first time concurrently analyzed paternal and maternal genetic variance in the Keriyans living in the Taklimakan Desert by genotyping 17 Y-STR loci and sequencing the complete control region of mtDNA.

The data from Y-STR in this study show that the Keriyans possess not only Asian (south and central Asia) specific NRY haplogroup H, T L, Q and N, but also European specific haplogroup R1a, J2a and I. European specific R1a has been observed in Central Asia and emerged from the Kurgan culture in Central Asia about 15,000 years ago and expanded there afterwards [39]. Higher frequency of R1a in Keriyan suggests that the Keriyan belong to a Central Asian population. In contrast, two dominant Y haplogroups D [40] and O [41] in Tibetans which are also Asian-specific were not observed in the Keriyans in this study, indicating that the modern Keriyans are not offspring of the Tibetans. It is also noted that the genetic distances between the Keriyans and the Uyghur from Xinjiang, or Siberia or Russians are much closer than the ones between the Keriyans and other populations including Chinese Han, Mongolian, and Tibetans. In addition to this, in the Network of NRY Haplotypes comprised of the selected 13 Y-STR loci from 14 populations, the majority of Keriyan individuals clustered together with the Uyghur, Russian and East Croatian, whereas a very small portion of the Keriyans were distributed among Mongolians. Haplotypes of Tibetans and Chinese Hans are separated from the Keriyans, indicating that the patrilineages of the Keriyan apparently do not have an affinity with Chinese Han and Tibetan. Taken together, we could conclude that the Keriyan is an admixture population of West and East Eurasian with West Eurasian prevalence in terms of patrilineages and male Tibetans are not a contributor of Y chromosome to the Keriyan population.

In addition to patrilineages, we also confirmed from their matrilineages that the Keriyan is a West-East Eurasian admixed population by analysis of the control regional sequences of the mtDNA. It is worthy of note that European maternal lineage makes relatively less contribution to the Keriyan than their patrilineages (50% vs. 82%), indicating a sex-bias in forming the Keriyan gene pool. In order to track the origin of matrilineages of the Keriyan, we compared the maternal genetic variance among the surrounding populations. As demonstrated by haplogroup patterns, Keriyan groups including our study here and another previously reported by Zhou [37] are closer to the Uyghur, Russian and Siberia populations than to Chinese Han, or Tibetans or Mongolians in genetics, which is in line with the patrilineage results.

East Asian specific mtDNA haplogroup M7c, M9 and M11 and North Asian haplogroup M8a frequently exist in the Keriyans with one or two step mutation from the ancestral roots. The above subgroup of haplogroup M arose in the Keriyan recently ∼3000–4000 years, much earlier than the Guge Kingdom collapse in 1630. This relative short coalescence time is consistent with the results from archeological and genetic studies on remains excavated from ancient tombs in the Tarim Basin. Except haplogroup M9a’b, the Keriyan does not share any sequences in the HV I segment with the Tibetans. Nevertheless, only two Tibetans shared the same sequences with the Keriyans in each of the two sub-haplotypes, while the majority of haplotypes of M9 in the Tibetans are not shared with the Keriyan or Daheyan populations. This further confirms that it is impossible that modern Keriyans are the descendants of ancient Guge Tibetans.

Moreover, we also found that low gene diversities on mtDNA and Y-STR loci in the Keriyan population reflect the isolation of the Keriyan population from other populations and less interaction with other populations. We have to admit that further analysis on NRY SNP will help address the coalescence time of Y chromosomal haplogroups since Y-STR only reflect recent mutations, not like SNP for ancient mutations.

The genetic distances between Keriyan and Uyghur both in Maternal and paternal lineages are nearly the shortest compared with the ones between the Keriyan and the other populations. Three Keriyans and one Uyghur from Xinjiang share the same haplotype comprised 17 Y-STR loci, indicating gene inflow from the adjacent Uyghur. Additionally, Keriyans share the same appearance, costume, language, written script, religion, and custom with the Uygurs in Yutian and Yutian is only 250 km away from the Keriyans. Taken together, we can’t rule out the hypothesis that the modern Keriyan is an off-spring of Uyghurs.

In conclusion, the Keriyan is an admixture population of Western and East Eurasian. There are a sex-bias on gene pool formation and human being migration of Keriyan. The Keriyans are not the descendants of the Tibetan tribe, but may originate from local Uyghur populations.

## Supporting Information

Table S1 **Pairwise Rst value calculated by AMOVA analysis offered by the YRDH website.**
(XLSX)

Table S2 **Mutations and frequencies of sub-haplogroups observed in Keriyan.**
(XLS)
